# The Spread of Southern Rice Black-Streaked Dwarf Virus Was Not Caused by Biological Changes in Vector *Sogatella furcifera*

**DOI:** 10.3390/microorganisms12061204

**Published:** 2024-06-14

**Authors:** Keiichiro Matsukura, Masaya Matsumura

**Affiliations:** 1 Institute for Plant Protection, National Agriculture and Food Research Organization (NARO), Tsukuba 305-0856, Ibaraki, Japan; 2 Koshi Research Station, Institute for Plant Protection, National Agriculture and Food Research Organization (NARO), Koshi 861-1192, Kumamoto, Japan

**Keywords:** Delphacidae, evolution, Fijivirus, planthopper, RT-qPCR, salivary gland, *Sogatella furcifera*

## Abstract

The pandemic of Southern rice black-streaked dwarf virus (SRBSDV) in and after the late 2000s caused serious yield losses in rice in Southeast and East Asia. This virus was first recorded in China in 2001, but its exclusive vector insect, *Sogatella furcifera*, occurred there before then. To clarify the evolutionary origin of SRBSDV as the first plant virus transmitted by *S. furcifera*, we tested virus transmission using three chronological strains of *S. furcifera*, two of which were established before the first report of SRBSDV. When the strains fed on SRBSDV-infected rice plants were transferred to healthy rice plants, those established in 1989 and 1999 transmitted the virus to rice similarly to the strain established in 2010. SRBSDV quantification by RT-qPCR confirmed virus accumulation in the salivary glands of all three strains. Therefore, SRBSDV transmission by *S. furcifera* was not caused by biological changes in the vector, but probably by the genetic change of the virus from a closely related *Fijivirus*, Rice black-streaked dwarf virus, as suggested by ecological and molecular biological comparisons between the two viruses. This result will help us to better understand the evolutionary relationship between plant viruses and their vector insects and to better manage viral disease in rice cropping in Asia.

## 1. Introduction

Southern rice black-streaked dwarf virus (SRBSDV) is a persistently propagative *Fijivirus* (family *Reoviridae*) that causes stunted growth and twisted leaf tips in rice (*Oryza sativa*) [1,2]. This virus was first detected in Guangdong Province, China, in 2001, and caused serious damage to rice production over a wide area of Southeast and East Asia in and after the late 2000s [3,4]. For example, a pandemic of SRBSDV in the late 2000s in China led to 30% to 50% yield losses of rice [2].

SRBSDV is transmitted exclusively by the white-backed planthopper, *Sogatella furcifera*, and it is the only plant virus transmitted by this planthopper [2]. The annual long-distance migration of *S. furcifera* from Southeast to East Asia [5] and resistance to insecticides [6] led to the horizontal spread of SRBSDV. SRBSDV-carrying *S. furcifera* live longer [7] and become more tolerant to heat stress [8] than virus-free *S. furcifera*. Moreover, SRBSDV infection alters the feeding preference of *S. furcifera* so that the virus is horizontally transmitted more efficiently [9]. These facts indicate that the viral infection during the rapid expansion of SRBSDV was not commensal only to the virus, but mutual between SRBSDV and *S. furcifera*. Although such an ecological interaction between SRBSDV and *S. furcifera* has been confirmed, the origin of SRBSDV is unclear. The evolution of insect-transmitted viruses is generally driven by a long history of plant–virus vector interactions under varied environmental conditions [10,11,12]. Therefore, we can expect that biological and ecological factors would have been involved in the appearance of SRBSDV as the first plant virus transmitted by *S. furcifera*. Clarifying the factors causing the spread of SRBSDV is important for preventing future epidemics of SRBSDV and other potential rice viruses transmitted by hemipteran pests.

The causal factors of insect-mediated plant virus transmission have been classified into four types according to a comprehensive review of pandemics and epidemics of plant viruses worldwide [13]. Two of them—the introduction of vulnerable cultivars and agricultural intensification—are caused by human activities aimed at increasing yields, whereas the other two factors occur in nature. One natural trigger are phenotypic changes in the virus due to genetic changes resulting from mutation and recombination. Phenotypic changes associated with virulence such as the evasion of the host’s immune system and adaptation to vector insects can lead to the appearance and epidemics of novel viral diseases [14,15]. The other natural, ecological trigger is the appearance of efficient vector insects [16]. Ecological and physiological changes in the population structure of vector insects often cause epidemics of viral disease. For example, the replacement of the native biotype JpL of *Bemisia tabaci* with biotypes B and Q in Japan as a result of their high resistance to various insecticides [17] promoted pandemics of Tomato yellow leaf curl virus and Cucurbit chlorotic yellows virus in horticultural crops [18,19]. Unlike in the case of phenotypic adaptations mediated by genetic changes in the virus itself, changes in the vector can lead to epidemics of viruses as important causes of disease before an epidemic.

Some phylogenetic and molecular biological works have suggested that SRBSDV has diverged from closely related rice viruses by means of mutation, recombination, or both. SRBSDV is phylogenetically close to Rice black-streaked dwarf virus (RBSDV), another rice virus, which is transmitted by the small brown planthopper, *Laodelphax striatellus* [20]. The typical symptoms, such as stunted growth and the appearance of white to black waxy galls, are similar in SRBSDV and RBSDV [21]. Furthermore, SRBSDV can accumulate in the gut of *L. striatellus*, although the titer is low [22]. These facts support the hypothesis that SRBSDV is a novel virus that was generated by genetic change from RBSDV (or another closely related *Fijivirus*) and succeeded in acquiring a novel insect vector. While the genetic change hypothesis is supported as a main stream of the origin of SRBSDV, other evidence suggests that biological changes in *S. furcifera* caused the pandemic: SRBSDV did not occur seriously until the late 2000s [3,4] despite its first detection in Guangdong Province in China in 2001 [1], meaning that the virus did not spread immediately after its appearance. In contrast, the occurrence status of *S. furcifera* in Southeast and East Asia changed markedly in the 2000s: the overuse of particular insecticides in these regions resulted in the appearance and spread of insecticide-resistant populations of *S. furcifera* [6]. The spread of the use of Chinese hybrid rice cultivars also promoted outbreaks of infestations by this planthopper, because these cultivars favor its reproduction [23]. Changes in the population structure of *S. furcifera* due to these artificial factors could have led to increased opportunities for SRBSDV transmission in the field, as has occurred with *B. tabaci* [17,18,19]. To examine whether the biological changes involving *S. furcifera* in Southeast and East Asia influenced the pandemic of SRBSDV in the late 2000s, we compared the transmission efficiency and accumulation of SRBSDV among three chronological strains of *S. furcifera*.

## 2. Materials and Methods

### 2.1. Virus and Insect Strains Used

The SRBSDV strain used was collected from a rice plant in Koshi, Kumamoto (32.88° N, 130.75° E) on 19 July 2013. It was maintained in a greenhouse using a method described previously [4].

Three strains of *S. furcifera* were used: WBPH1989, WBPH1999, and WBPH2010. The first two strains were established from field populations collected before the first appearance of SRBSDV in 2001, and the third was established after the pandemic of SRBSDV. WBPH1989 was collected from rice paddy fields in Chikugo, Fukuoka, Japan (33.02° N, 130.49° E), in 1989. WBPH1999 and WBPH2010 were collected from rice paddy fields in Koshi, Kumamoto, Japan (32.87° N, 130.73° E), in 1999 and 2010, respectively. Since their collection, our laboratory has kept these age-representative strains by mass-rearing on rice seedlings [24] at 25 °C under a 16 h light/8 h dark photoperiod to compare and evaluate their biological potential as a rice pest among different ages. In fact, Myint et al. [25] found that WBPH1989 is virulent to a rice variety carrying a resistant gene *Wbph1* but avirulent to another variety carrying a resistant gene *Wbph2*, whereas WBPH1999 can feed on both resistant varieties, and reached the significant conclusion that *S. furcifera* broke *Wbph2* between 1989 and 1999. In addition, to confirm the biological divergence among three strains, we preliminarily examined the median lethal dose (LD_50_) of WBPH1989 and WBPH1999 against fipronil using an established topical application method [26] in 2005–2006, and compared the published LD_50_ of WBPH2010 [26]. WBPH1989 showed the lowest LD_50_ (0.062, at 24 h after treatment), followed by WBPH1999 (2.3) and WBPH2010 (3.9), reflecting a field situation that fipronil appeared on the market as an insecticide in the mid-1990s. These diverged biological characteristics as rice pests support an applicability of these three strains to compare the transmission efficiency of SRBSDV in *S. furcifera* among different ages.

We also used *L. striatellus* and the brown planthopper *Nilaparvata lugens* to determine the efficiency of SRBSDV transmission by other rice planthoppers; *L. striatellus* was collected from grass weeds in a rice field in Yame, Fukuoka (33.24° N, 130.67° E), in 2009, and *N. lugens* was collected from rice paddy fields in Koshi, Kumamoto (32.87° N, 130.73° E), in 2010.

All planthopper strains used were mass-reared on rice seedlings (‘Reiho’) at 25 °C under a 16 h light/8 h dark photoperiod as described by us previously [24]. Only adult males were used in the experiments, because females produce large numbers of eggs that may induce resistance to the viral infection of rice [27,28]; moreover, hatchlings cause serious (and sometimes lethal) damage to infected rice plants.

### 2.2. SRBSDV Infection of Rice Seedlings by Three Strains of S. furcifera

SRBSDV acquisition and transmission were performed by following established methods in the laboratory [4,29]. Approximately 100 newly emerged adult males of WBPH1989, WBPH1999, and WBPH2010 were released onto SRBSDV-infected rice plants at the early tillering stage (one plant used for each strain) that were covered with an acrylic cage with nylon gauze for ventilation. Five days after the males had been released to acquire the virus, they were transferred to healthy young rice seedlings (‘Reiho’) at a density of three adults per seedling (20 seedlings per strain). The seedlings were individually covered with an acrylic cage with nylon gauze and kept at 25 °C under a 16 h light/8 h dark photoperiod for 28 days. After the males had been kept with the rice plants for 7 days for virus inoculation, they were removed to avoid additional damage from feeding. At the end of this virus inoculation period, the SRBSDV infection of each rice seedling was confirmed by RT-qPCR ([4]) as described in Section 2.3.

RT-qPCR was also used for SRBSDV quantification in the insects’ salivary glands and other body parts. Ten adult males of each strain that had been released on SRBSDV-infected rice for 5 days to acquire the virus were used. The salivary glands of each insect were dissected out under the microscope, and the salivary glands and the remaining body parts (i.e., the whole body minus the salivary glands) were used for virus quantification by RT-qPCR.

### 2.3. SRBSDV Extraction, Detection, and Quantification by RT-qPCR

A rice leaf sheath (~50 mg) was soaked in a mixture of 250 µL of TriPure Isolation Reagent (Roche Diagnostics Co., Ltd., Tokyo, Japan) and 50 µL of chloroform. Individual salivary glands or whole body parts minus the salivary glands were added to each extract. The samples were crushed with a Multibeads Shocker (Yasui Kikai Co., Ltd., Osaka, Japan) at 2000 rpm for 30 s. After the centrifugation of each sample at 6500× *g* for 10 min, the supernatant was transferred to a new tube and an equal amount of isopropanol was added. After centrifugation at 20,000× *g* for 15 min, the precipitate was rinsed with 300 µL of 80% ethanol. After vacuum drying, the precipitate was resuspended in 25 µL of distilled water.

The SRBSDV titer in each extract was quantified on a LightCycler 480FLM PCR platform (Roche Diagnostics Co., Ltd.) with a TaKaRa Onestep SYBR PrimeScript RT-PCR kit (Takara Bio Co., Ltd., Kusatsu, Japan) in accordance with the manufacturer’s protocols. A primer set (forward: 5′-gag cgg agt ctc ctc att ta-3′; reverse: 5′-gca acg atg aac ctt tct ct-3′) designed from the capsid protein gene of SRBSDV [4] was used for the specific amplification of SRBSDV. Rice samples with <30 threshold cycles (Ct) for the detection of amplified fragments were regarded as infected [4]. Similarly, the titer of the target RNA (i.e., the number of target RNA copies per specimen) in planthopper specimens was determined in the range of 11 to 30 Ct by using a calibration curve established in our previous report [29]. The titer determined was corrected on the basis of *actin* gene expression determined by RT-qPCR using a primer set (forward: 5′-ccg gta ttg tgc tcg act cc-3′, reverse: 5′-gct gtg gcc att tcc tgt tc-3′) common to all three planthoppers used in the study [29].

### 2.4. SRBSDV Transmission by L. striatellus and N. lugens

The virulence of *L. striatellus* and *N. lugens* was compared with *S. furcifera* strain WBPH2010 as a control. A hundred newly emerged adult males were released onto an SRBSDV-infected rice plant (one plant used for each species) for seven days and then transferred to young rice seedlings, each covered with an acrylic cage with nylon gauze (one adult per seedling). The adults were removed on day 7, and the seedlings were then kept at 25 °C under a 16 h light/8 h dark photoperiod for 25 days for virus replication. Infection with SRBSDV was examined by RT-qPCR as described above. We tested 50 *L. striatellus*, 10 *N. lugens*, and 40 *S. furcifera* (controls).

### 2.5. Comparison of SRBSDV Accumulation among Rice Planthoppers

SRBSDV accumulation was compared among adult males of *S. furcifera* (WBPH2010), *L. striatellus*, and *N. lugens*. Thirty adult males of each species were released onto a SRBSDV-infected rice plant (one plant used for each species), each covered with an acrylic cage with nylon gauze. At 2 and 10 days after their release, 8 males of each species were collected from the plants and used for SRBSDV quantification.

Virus titers were quantified by RT-qPCR as described above and correction against *actin*. To compare *actin* gene expression among planthopper species, the average expression in each planthopper was determined as follows. The whole body of newly emerged adult male (*n* = 12 for each species) was soaked in a mixture of 250 µL TriPure Isolation Reagent and 50 µL of chloroform. After the addition of 2 µL of SRBSDV extract from infected rice (see Section 2.3) as an internal standard, the specimens were crushed with a pestle. RNA was purified by centrifugation and the addition of 80% ethanol (as above), and the final precipitate was resuspended in 10 µL of distilled water. Concentrations of the *actin* gene and SRBSDV were determined by RT-qPCR, and the relative *actin* gene expression was calculated as:

(1) $$Relative\;actin\;gene\;expression=2^{\left(Ct\;of\;SRBSDV\;-\;Ct\;of\;Actin\right)}$$

To simplify the correction, the efficiencies of SRBSDV and *actin* gene amplification in RT-qPCR were both fixed at 2. Finally, the ratios of the *actin* gene expression of *L. striatellus* and *N. lugens* to that of *S. furcifera* were determined.

### 2.6. Statistical Analyses

The absolute and relative titers of SRBSDV and the relative *actin* gene expression among strains of *S. furcifera* and among species were tested using Tukey’s HSD test. Because our data contained some virus titers of 0 (i.e., the virus was not detected by RT-qPCR), all titer data were adjusted by adding 1 (copy per sample). Both adjusted and relative titers were log-transformed before statistical testing. The rates of transmission of SRBSDV to rice seedlings among *S. furcifera* strains and among species were compared using Fisher’s exact test with Holm–Bonferroni correction for multiple comparison. All statistical analyses were performed in SciPy v. 1.11.4 in Python 3.9.10 software.

## 3. Results

### 3.1. SRBSDV Transmission by S. furcifera Collected in Different Years

*Sogatella furcifera* strains WBPH1989 and WBPH1999, which were collected before the pandemic of SRBSDV in the late 2000s, transmitted SRBSDV to rice at a rate not significantly different from that of the strain collected in 2010 (Fisher’s exact test with Holm–Bonferroni correction, α = 0.05; Table 1).

**Figure 1:**
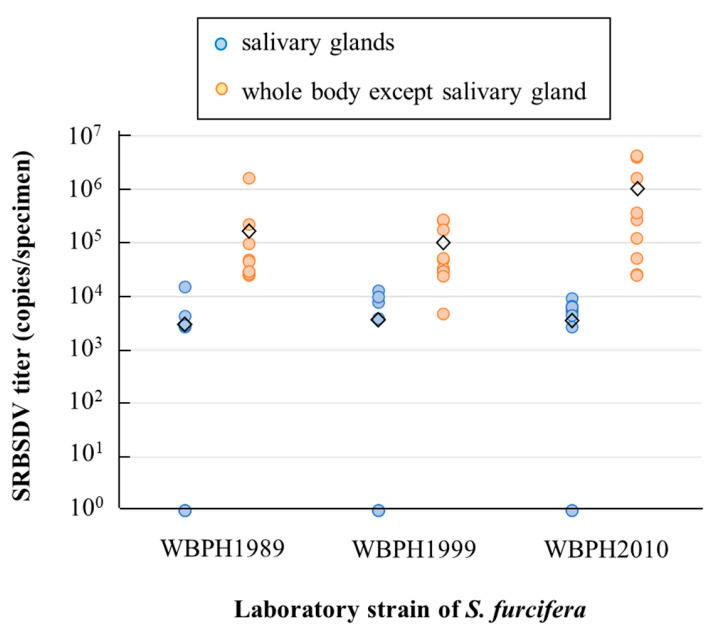
Accumulation of SRBSDV in the salivary glands and remaining body parts of the three chronological strains of *Sogatella furcifera*. ◇ Mean virus titers in each treatment. No significant difference in virus titers within the same body parts was detected among *S. furcifera* strains (Tukey’s HSD test, α = 0.05).

SRBSDV accumulation in both the salivary glands and the remaining body parts did not significantly differ among the three *S. furcifera* strains either (Tukey’s HSD test after log-transformation, α = 0.05; Figure 1). The average SRBSDV titer in the salivary glands was 3854 copies per sample, and that in the remainder of the body was 463,664 copies per sample.

### 3.2. SRBSDV Transmission by Other Rice Planthoppers

No SRBSDV transmission by *L. striatellus* (*n* = 50) or *N. lugens* (*n* = 10) was observed, whereas 87.5% of *S. furcifera* (*n* = 40) transmitted the virus under the same experimental conditions (Table 2), with a significant difference (Tukey’s HSD test after log-transformation, α = 0.05).

The relative amounts of *actin* gene expression significantly differed among the whole bodies of adult males of the three planthoppers (Table 3). Gene expression was significantly higher in the whole bodies of *S. furcifera* and *N. lugens* than in that of *L. striatellus* (Tukey’s HSD test after log-transformation, α = 0.05). The magnifications of *actin* gene expression relative to that in *S. furcifera* were 0.026 in *L. striatellus* and 1.308 in *N. lugens*.

**Figure 2:**
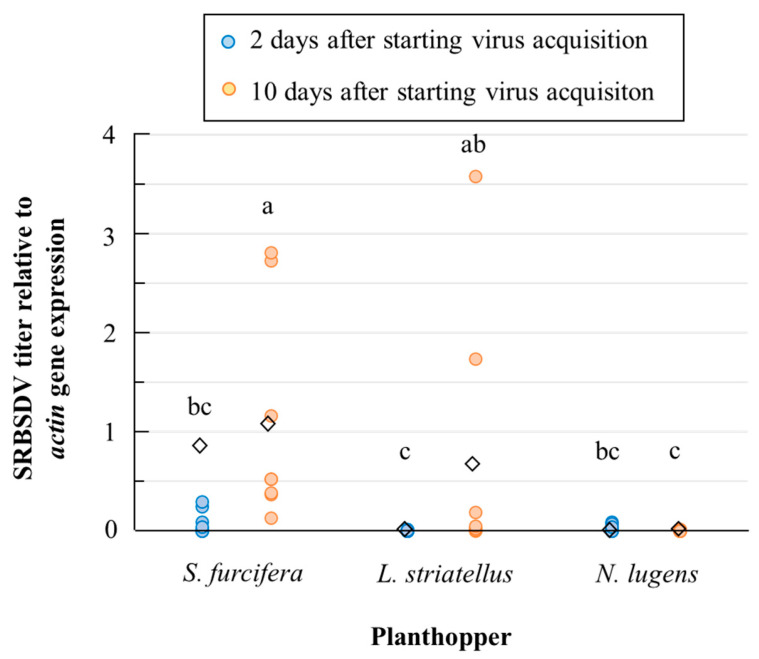
SRBSDV titers relative to *actin* gene expression in the whole bodies of three rice planthopper species fed on SRBSDV-infected rice. ◇ Mean relative virus titers in each treatment. Varied *actin* gene expression among the planthopper species was corrected by the relative *actin* gene expression per specimen, shown in Table 3. Data points with the same letter are not significantly different (Tukey’s HSD test, α = 0.05).

The relative SRBSDV titers corrected by the magnification of *actin* gene expression specific to each planthopper species significantly varied among species and durations of virus acquisition in some instances (Figure 2). The relative SRBSDV titer in the whole body of *S. furcifera* after 10 days of virus acquisition was significantly higher than those in the other species at each treatment duration, except in the case of *L. striatellus* with a virus acquisition period of 10 days (Tukey’s HSD test after log-transformation, α = 0.05). The virus titer increased significantly between 2 and 10 days of virus acquisition in *S. furcifera* and *L. striatellus*, whereas titers were very low in *N. lugens* at both 2 and 10 days.

## 4. Discussion

All chronological strains of *S. furcifera*, including those that were established before the first report of SRBSDV in 2001, transmitted SRBSDV to rice (Table 1) and accumulated SRBSDV in their salivary glands and other body parts (Figure 1), which is the determinant step for persistent propagative plant virus to be mediated by the vector [30]. The successful SRBSDV transmission by WBPH1989 and WBPH1999 is not a complete proof of the potential SRBSDV transmission ability of *S. furcifera* before the pandemic of this virus because we cannot exclude the possibility that these strains acquired the virus transmission ability during mass-rearing in laboratory for long years. Nevertheless, the fact that both WBPH1989 and WBPH1999 strains transmitted the virus despite remaining biological characteristics such as virulence to resistant varieties [25] and insecticide susceptibility [26] (also see Section 2.1) at established ages from field is strong evidence that *S. furcifera* had the ability to transmit SRBSDV before the first detection of this virus in 2001. *Sogatella furcifera* was a serious pest of rice in Southeast and East Asia even before the SRBSDV pandemic in the 2000s [23,31], and it has frequently exchanged genes among geographical populations [32] through annual long-distance migrations [5]. The pandemic of SRBSDV would have occurred earlier if this virus had existed in the 1980s and the 1990s. Our results and these facts indicate that the pandemic of SRBSDV in Southeast and East Asia in the late 2000s was not caused by biological changes related to the ability of *S. furcifera* to transmit the virus, but instead it was caused by the appearance of novel virus in about 2000. As suggested by fragmentary evidence [21,22], the adaptation of SRBSDV to a novel vector, *S. furcifera*, through genetic change from a closely related virus was likely a primary causative factor of the viral pandemic.

SRBSDV is the first plant virus to be transmitted by *S. furcifera*. Assuming that this virus diverged from RBSDV or another closely related *Fijivirus*, the clarification of the process of the evolutionary physiological adaptation of SRBSDV to the novel vector is important. The relationship between rice reoviruses and vector insects is species-specific, because the viruses need to break the physical barriers of the vector insects and disrupt their immune systems [33]. Our results support previous findings that SRBSDV accumulates in the whole body of *L. striatellus* (Figure 2) but is not transmitted by it (Table 2) [22], because SRBSDV cannot disrupt the immune system of *L. striatellus* to enter the hemolymph and other body organs from the gut [34]. Some studies have addressed the physiological mechanism of SRBSDV spread into the body organs of *S. furcifera*. SRBSDV infecting cells of the insect vector spreads to neighboring cells or organs by inducing protein tubules, which allow the virus particles to pass physical barriers such as the cell wall and basal lamina along actin-based filopodia [33,35]. SRBSDV infection in *S. furcifera* also induces incomplete autophagy by blocking autophagosome–lysosome fusion, thus helping the virus to evade the immune system of the vector [36]. Frequent recombination is considered a factor driving the evolution of SRBSDV [37]. However, the molecular origin of the complex and specific adaptation of SRBSDV to the defense system of *S. furcifera* is unclear, because SRBSDV is the first plant virus to be transmitted by this planthopper. Further physiological and molecular biological studies are needed to clarify the process of adaptation of SRBSDV to *S. furcifera*.

The occurrence of SRBSDV is continuing to expand. The occurrence of SRBSDV-infected rice was reported in southwestern India in 2022 [38]. The first detection of the virus in Jiansu Province, China, was reported in 2023 [39]. The continuous expansion of SRBSDV infection together with outbreaks of *S. furcifera* might have been caused by the appearance of this first ‘symbiotic’ virus in *S. furcifera*. Virus acquisition by insect vectors has both positive and negative impacts on the survival and reproduction of the virus and vector [11]. Several ecological factors, such as the development of insecticide resistance [6], a shift to *S. furcifera*-susceptible cultivars [23], and the expansion of the overwintering range due to global warming [40], could increase the outbreaks of *S. furcifera*. In addition, SRBSDV acquisition has benefited both *S. furcifera* and SRBSDV in laboratory tests [7,8,9]. The effects of this symbiosis on the outbreaks and range expansion of *S. furcifera* and SRBSDV in the field should be clarified to improve our understanding of the evolution of insect-transmitted plant viruses.

Our finding that SRBSDV acquired *S. furcifera* as a novel vector without any biological changes related to the transmission of the virus indicates the importance of vector management to prevent further expansion of SRBSDV infection. Modeling approaches to predict the long-distance migration of *S. furcifera* from weather conditions [5,41] and to evaluate the risk of virus expansion from the vector density and virus acquisition rate [42] are important for decision-making concerning insecticide application. In addition, the immediate detection of SRBSDV-infected rice in the field by using RT-PCR [1], RT-qPCR [28], and ELISA [43], and the breeding of SRBSDV-resistant rice [44] will contribute to better preventive control of this viral disease in Asian rice cropping systems.

## Figures and Tables

**Table 1:** Transmission rates of Southern rice black-streaked dwarf virus by adult males of the three laboratory strains of *Sogatella furcifera* collected in different years.

Year of Collection	Number of Rice Seedlings Tested	Transmission Rate (%) ^1^
1989	20	40
1999	20	20
2010	20	15

^1^ No significant pairwise differences in transmission rates were detected between any pairs of *S. furcifera* strains (Fisher’s exact test with Holm–Bonferroni correction, α = 0.05).

**Table 2:** Transmission rates of Southern rice black streaked dwarf virus to rice seedlings by rice planthoppers.

Species	Number of Insects Tested	Transmission Rate ^1^ (%)
*Sogatella furcifera*	40	87.5 ^a^
*Laodelphax striatellus*	50	0 ^b^
*Nilaparvata lugens*	10	0 ^b^

^1^ Values with the same letter are not significantly different among species (Fisher’s exact test with Holm–Bonferroni correction, α = 0.05).

**Table 3:** Comparison of actin gene expression in whole bodies of adult males of three rice planthoppers.

Species	Number of Specimens Tested	Relative Actin Gene Expression Per Specimen ^1^ (±SE)	Ratio
*Sogatella furcifera*	12	3.83 ± 0.54 ^a^	1
*Laodelphax striatellus*	12	0.10 ± 0.03 ^b^	0.03
*Nilaparvata lugens*	12	5.01 ± 1.03 ^a^	1.31

^1^ Values with the same letter are not significantly different among species (Tukey’s HSD test, α = 0.05).

## Data Availability

All the data associated with this manuscript are provided within the manuscript.

## References

[1] Zhou G.H., Wen J.J., Cai D.J., Li P., Xu D.L., Zhang S.G. (2008). Southern rice black-streaked dwarf virus: A new proposed *Fijivirus* species in the family Reoviridae. Chin. Sci. Bull..

[2] Lv M.-F., Xie L., Wang H.-F., Wang H.-D., Chen J.-P., Zhang H.-M. (2017). Biology of Southern rice black-streaked dwarf virus: A novel fijivirus emerging in East Asia. Plant Pathol..

[3] Hoang A.T., Zhang H.M., Yang J., Chen J.P., Hébrard E., Zhou G.H., Vinh V.N., Cheng J.A. (2011). Identification, characterization, and distribution of southern rice black-streaked dwarf virus in Vietnam. Plant Dis..

[4] Matsukura K., Towata T., Sakai J., Onuki M., Okuda M., Matsumura M. (2013). Dynamics of Southern rice black-streaked dwarf virus in rice and implication for virus acquisition. Phytopathology.

[5] Otuka A., Watanabe T., Suzuki Y., Matsumura M., Furuno A., Chino M. (2005). Real-time prediction system for migration of rice planthoppers *Sogatella furcifera* (Horváth) and *Nilaparvata lugens* (Stål) (Homoptera: Delphacidae). Appl. Entomol. Zool..

[6] Matsumura M., Sanada-Morimura S., Otuka A., Sonoda S., Thanh D.V., Chien H.V., Tuong P.V., Loc P.M., Liu Z.-W., Zhu Z.R. (2018). Insecticide susceptibilities of the two rice planthoppers *Nilaparvata lugens* and *Sogatella furcifera* in East Asia, the Red River Delta, and the Mekong Delta. Pest Manag. Sci..

[7] Zhang J., Zheng X., Chen Y., Hu J., Dong J., Su X., Zhang Z. (2014). Southern rice black-streaked dwarf virus infection improves host suitability for its insect vector, *Sogatella furcifera* (Hemiptera: Delphacidae). J. Econ. Entomol..

[8] Xu D., Zhong T., Feng W., Zhou G. (2016). Tolerance and responsive gene expression of *Sogatella furcifera* under extreme temperature stresses are altered by its vectored plant virus. Sci. Rep..

[9] Wang H., Xu D., Pu L., Zhou G. (2014). Southern rice black-streaked dwarf virus alters insect vectors’ host orientation preferences to enhance spread and increase rice ragged stunt virus co-infection. Phytopathology.

[10] Ng J.C.K., Falk B.W. (2006). Virus-vector interactions mediating nonpersistent and semipersistent transmission of plant viruses. Ann. Rev. Phytopathol..

[11] Eigenbrode S.D., Bosque-Pérez N.A., Davis T.S. (2018). Insect-borne plant pathogens and their vectors: Ecology, evolution, and complex interactions. Ann. Rev. Phytopathol..

[12] Islam W., Noman A., Naveed H., Alamri S.A., Hashem M., Huang Z., Chen H.Y.H. (2020). Plant-insect vector-virus interactions under environmental change. Sci. Total Environ..

[13] Jones R.A.C. (2021). Global plant virus disease pandemics and epidemics. Plants.

[14] Martin D.B., Biagini P., Lefeuvre P., Golden M., Roumagnac P., Varsani A. (2011). Recombination in eukaryotic single stranded DNA viruses. Viruses.

[15] Simon-Loriere E., Holmes E.C. (2011). Why do RNA viruses recombine?. Nat. Rev. Microbiol..

[16] Girbertson R.L., Batuman O., Webster C.G., Adkins S. (2015). Role of the insect supervectors *Bemisia tabaci* and *Frankliniella occidentalis* in the emergence and global spread of plant viruses. Ann. Rev. Virol..

[17] Horowitz A.R., Ghanim M., Roditakis E., Nauen R., Ishaaya I. (2020). Insecticide resistance and its management in *Bemisia tabaci* species. J. Pest. Sci..

[18] Ueda S., Brown J.K. (2006). First report of the Q biotype of *Bemisia tabaci* in Japan by mitochondrial *cytochrome oxidase* I sequence analysis. Phytoparasitica.

[19] Okuda M., Okazaki S., Yamasaki S., Okuda S., Sugiyama M. (2010). Host range and complete genome sequence of *Cucurbit chlorotic yellows virus*, a new member of the genus Crinivirus. Phytopathology.

[20] Matsukura K., Sanada-Morimura S., Fujii T., Matsumura M. (2019). Potential risks of poaceous plants as infectious sources of *Rice black-streaked dwarf virus* transmitted by the small brown planthopper, *Laodelphax striatellus*. Plant Dis..

[21] Cuong H.V., Hai N.V., Man V.T., Matsumoto M. (2009). Rice dwarf disease in North Vietnam in 2009 is cause by southern rice black-streaked dwarf virus (SRBSDV). Bull. Inst. Trop. Agric. Kyushu Univ..

[22] Hajano J.U.D., Wang B., Ren Y., Lu C., Wang X. (2015). Quantification of southern rice black streaked dwarf virus and rice black streaked dwarf virus in the organs of their vector and nonvector insect over time. Virus. Res..

[23] Sogawa K., Heong K., Cheng J., Escalada M. (2015). Planthopper outbreaks in different paddy ecosystems in Asia: Man-made hopper plagues that threatened the green revolution in rice. Rice Planthoppers.

[24] Matsumura M., Takeuchi H., Satoh M., Sanada-Morimura S., Otuka A., Watanabe T., Thanh D.V. (2008). Species-specific insecticide resistance to imidacloprid and fipronil in the rice planthoppers and *Sogatella furcifera* in East and South-east Asia. Pest Manag. Sci..

[25] Myint K.K.M., Yasui H., Takagi M., Matsumura M. (2009). Virulence of long-term laboratory populations of the brown planthopper, *Nilaparvata lugens* (Stål), and whitebacked planthopper, *Sogatella furcifera* (Horváth) (Homoptera: Delphacidae), on rice differential varieties. Appl. Entomol. Zool..

[26] Matsumura M., Sanada-Morimura S., Otuka A., Ohtsu R., Sakumoto S., Takeuchi H., Satoh M. (2014). Insecticide susceptibilities in populations of two rice planthoppers, *Nilaparvata lugens* and *Sogatella furcifera*, immigrating into Japan in the period 2005–2012. Pest Manag. Sci..

[27] Seino Y., Suzuki Y., Sogawa K. (1996). An ovicidal substance produced by rice plants in response to oviposition by the whitebacked planthopper, *Sogatella furcifera* (HORVÁTH) (Homoptera: Delphacidae). Appl. Entomol. Zool..

[28] Suzuki Y., Sogawa K., Seino Y. (1996). Ovicidal reaction of rice plants against the whitebacked planthopper, *Sogatella furclfera* HORVÁTH (Homoptera: Delphacidae). Appl. Entomol. Zool..

[29] Matsukura K., Towata T., Yoshida K., Sakai J., Okuda M., Onuki M., Matsumura M. (2015). Quantitative analysis of Southern rice black-streaked dwarf virus in Sogatella furcifera and virus threshold for transmission. Phytopathology.

[30] Hogenhout S.A., Ammar E.D., Whitefield A.E., Redinbaugh M.G. (2008). Insect vector interactions with persistently transmitted viruses. Ann. Rev. Phtyopathol..

[31] Cheng J., Heong K., Cheng J., Escalada M. (2015). Rice planthoppers in the past half century in China. Rice Planthoppers.

[32] Yang N., Dong Z., Chen A., Yin Y., Li X., Chu D. (2020). Migration of *Sogatella furcifera* between the greater Mekong subregion and northern China revealed by mtDNA and SNP. BMC Evol. Biol..

[33] Wei T., Li Y. (2016). Rice reovirus in insect vectors. Ann. Rev. Phytopathol..

[34] Jia D., Chen H., Mao Q., Liu Q., Wei T. (2012). Restriction of viral dissemination from the midgut determines incompetence of small brown planthopper as a vector of Southern rice black-streaked dwarf virus. Virus Res..

[35] Jia D., Mao Q., Chen H., Wang A., Liu Y., Wang H., Xie L., Wei T. (2014). Virus-induced tubule: A vehicle for rapid spread of virions through basal lamina from midgut epithelium in the insect vector. J. Virol..

[36] Zhang L., Liu W., Wu N., Wang H., Zhang Z., Liu Y., Wang X. (2023). Southern rice black-streaked dwarf virus induces incomplete autophagy for persistence in gut epithelial cells of its vector insect. PLoS Pathog..

[37] Xue J., Zhang H.-M., Yang J., Lv M.-F., Gao B.-D., Chen J.-P. (2013). Molecular characterization of Southern rice blacked-streaked dwarf virus (SRBSDV) from Vietnam. J. Phytopathol..

[38] Sharma S.K., Ghosh A., Gupta N., Singh A.K., Diksha D., Thapa P., Jangra S., Baranwal V.K. (2022). Evidence for association of southern rice black-streaked dwarf virus with the recently emerged stunting disease of rice in North-West India. Indian J. Genet. Plant Breed..

[39] Li C., Yang W., Zhang Y., Zhu F., Qiu Y., Du L., Lin F., Lan Y., Xu K., Zhou T. (2024). Investigation and characterization of rice dwarfing epidemic caused by southern rice black-streaked dwarf virus in Jiangsu in 2023. Virology.

[40] Hu S.-J., Fu D.-Y., Han Z.-L., Ye H. (2015). Density, demography, and influential environmental factors on overwintering populations of *Sogatella furcifera* (Hemiptera: Delphacidae) in southern Yunnan, China. J. Insect Sci..

[41] Hu S.-J., Sun S.-S., Fu D.-Y., Lü J.-P., Wang X.-Y., Yu Y.-P., Dong L.-M., Chen S.-Y., Ye H. (2020). Migration sources and pathways of the pest species *Sogatella furcifera* in Yunnan, China, and across the border inferred from DNA and wind analyses. Ecol. Evol..

[42] Matsukura K., Watanabe T., Matsumura M. (2017). 2017 An epidemic model of a rice virus transmitted by a migratory planthopper. J. Pest Sci..

[43] Wang Z., Yu D., Li X., Zeng M., Chen Z., Bi L., Liu J., Jin L., Hu D., Yang S. (2012). The development and application of a dot-ELISA assay for diagnosis of southern rice black-streaked dwarf disease in the field. Viruses.

[44] Zhou S., Zhao Y., Liang Z., Wu R., Chen B., Zhang T., Yang X., Zhou G. (2021). Resistance evaluation of dominant varieties against Southern rice black-streaked dwarf virus in southern China. Viruses.

